# Enhanced Chondrogenic Differentiation Activities in Human Bone Marrow Aspirates via *sox9* Overexpression Mediated by pNaSS-Grafted PCL Film-Guided rAAV Gene Transfer

**DOI:** 10.3390/pharmaceutics12030280

**Published:** 2020-03-21

**Authors:** Jagadeesh K. Venkatesan, Weikun Meng, Ana Rey-Rico, Gertrud Schmitt, Susanne Speicher-Mentges, Céline Falentin-Daudré, Amélie Leroux, Henning Madry, Véronique Migonney, Magali Cucchiarini

**Affiliations:** 1 Center of Experimental Orthopaedics, Saarland University Medical Center, D-66421 Homburg/Saar, Germany; jegadish.venki@gmail.com (J.K.V.); weikun.m@gmail.com (W.M.); ana.rey.rico@udc.es (A.R.-R.); schmitt_gertrud@web.de (G.S.); Susanne.Speicher-Mentges@uks.eu (S.S.-M.); henning.madry@uks.eu (H.M.); 2 LBPS/CSPBAT UMR CNRS 7244, Université Sorbonne Paris Nord, F-93430 Villetaneuse, France; falentin-daudre@univ-paris13.fr (C.F.-D.); amelie.leroux@univ-paris13.fr (A.L.); veronique.migonney@univ-paris13.fr (V.M.); 3 Department of Orthopaedic Surgery, Saarland University Medical Center, D-66421 Homburg/Saar, Germany

**Keywords:** cartilage repair, human bone marrow aspirates, rAAV, SOX9, pNaSS-grafted PCL films, chondrogenesis

## Abstract

Background: The delivery of therapeutic genes in sites of articular cartilage lesions using non-invasive, scaffold-guided gene therapy procedures is a promising approach to stimulate cartilage repair while protecting the cargos from detrimental immune responses, particularly when targeting chondroreparative bone marrow-derived mesenchymal stromal cells in a natural microenvironment like marrow aspirates. Methods: Here, we evaluated the benefits of providing a sequence for the cartilage-specific sex-determining region Y-type high-mobility group box 9 (SOX9) transcription factor to human marrow aspirates via recombinant adeno-associated virus (rAAV) vectors delivered by poly(ε-caprolactone) (PCL) films functionalized via grafting with poly(sodium styrene sulfonate) (pNaSS) to enhance the marrow chondrogenic potential over time. Results: Effective *sox9* overexpression was observed in aspirates treated with pNaSS-grafted or ungrafted PCL films coated with the candidate rAAV-FLAG-h*sox9* (FLAG-tagged rAAV vector carrying a human *sox9* gene sequence) vector for at least 21 days relative to other conditions (pNaSS-grafted and ungrafted PCL films without vector coating). Overexpression of *sox9* via rAAV *sox9*/pNaSS-grafted or ungrafted PCL films led to increased biological and chondrogenic differentiation activities (matrix deposition) in the aspirates while containing premature osteogenesis and hypertrophy without impacting cell proliferation, with more potent effects noted when using pNaSS-grafted films. Conclusions: These findings show the benefits of targeting patients’ bone marrow via PCL film-guided therapeutic rAAV (*sox9*) delivery as an off-the-shelf system for future strategies to enhance cartilage repair in translational applications.

## 1. Introduction

Focal cartilage defects are critical problems in clinical orthopaedics since the adult articular cartilage has a limited ability for full, self-repair as a result of an absence of access to regenerative cells due a lack of vascularization [1,2]. In addition, none of the current clinical options that include microfracture and cell transplantation are capable of stimulating the production of the natural (hyaline) cartilage in sites of injuries, leading to the formation of a fibrocartilaginous repair tissue made of type-I collagen instead of type-II collagen and proteoglycans, with poor mechanical properties and that may progress to generalized osteoarthritis [1,2,3,4]. Application of chondrogenically competent mesenchymal stromal cells (MSCs) [5,6,7] in focal cartilage lesions is an attractive approach to locally enhance the cartilage repair activities in a natural bone marrow environment as afforded when using marrow concentrates [8,9]. Still, even with such minimally invasive procedures, the repair tissue in the treated defects remains of poor structural and mechanical quality [8,9], showing that more effective, new treatments are needed for improved healing properties.

Biomaterial-guided gene transfer recently became the focus of translational cartilage research as a powerful tool to improve cartilage repair upon controlled delivery of gene shuttles to extend the expression and consequently the effects of the transgene products being applied [10,11,12], with reports showing the possibility of releasing nonviral [13,14,15,16,17,18,19] or lentiviral vectors [20,21,22,23]. In contrast to such gene vehicles that permit only transient gene expression (nonviral vectors) or carry the risk for oncogene activation upon integration in the host genome (lentiviral vectors), recombinant adeno-associated virus (rAAV) vectors may provide more effective and safer tools for cartilage repair and may be amenable to controlled delivery via scaffold-assisted procedures that may further protect the rAAV particles from neutralization by humoral immune responses [24], a problem that has precluded direct rAAV therapy in clinical applications [10,11].

As a matter of fact, a number of studies reported the potential of applying rAAV for experimental cartilage research via hydrogel systems (alginate, fibrin, poloxamers/poloxamines, self-assembling peptides, polypseudorotaxanes) [25,26,27,28,29,30,31,32,33] while there is still little information on the potential benefits of solid, mechanically more stable biomaterials that may provide scaffolding and stability to the target cells [34] for rAAV-mediated gene transfer. In this regard, we recently provided evidence that biocompatible solid polyester poly(ε-caprolactone) (PCL) [35], an aliphatic polyester approved by the FDA [36,37], further grafted with poly(sodium styrene sulfonate) (pNaSS) to activate reparative cellular responses [38] is capable of supporting the delivery of reporter rAAV gene vectors to effectively modify human bone marrow aspirates [39]. The goal of the present study was therefore to further test the ability of pNaSS-grafted PCL films to directly deliver a therapeutic rAAV vector coding for the cartilage-specific sex-determining region Y-type high mobility group box 9 (SOX9) transcription factor [40] in marrow aspirates as a means to trigger the chondroreparative activities in these samples in light of our earlier work showing that rAAV SOX9 can restore the extracellular matrix in human osteoarthritic cartilage [41]. Such a direct PCL-guided rAAV gene transfer approach may further improve the chondrogenic effects of rAAV SOX9 in marrow aspirates either via direct (free) vector gene transfer in the absence of PCL scaffold [42] or following seeding of initially SOX9-modified aspirates in the scaffolds [43]. SOX9 is indeed a strong candidate for cartilage repair as it is the essential, master regulator of cartilage formation [40,44] over other SOX family members [45] by directly activating chondrogenic genes such as type-II collagen and aggrecan [46,47].

Our data show that pNaSS-grafted PCL films are able to successfully promote the transfer and overexpression of the candidate rAAV *sox9* vector in human marrow aspirates for at least 21 days (the longest time point evaluated) relative to other treatments (rAAV-coated ungrafted PCL films and pNaSS-grafted or ungrafted PCL films without vector coating since rAAV does not alter the competence of the aspirates upon PCL-guided delivery) [39]. This led to enhanced biological activities and chondrogenic differentiation events (matrix deposition) in the *sox9*-treated aspirates *versus* conditions lacking *sox9*, independently from cell proliferative activities, and to a reduction of undesirable osteogenic and hypertrophic differentiation outcomes, especially when providing rAAV *sox9* via pNaSS-grafted PCL films. The current findings demonstrate the potential of PCL-assisted therapeutic rAAV gene transfer for applications to treat articular cartilage lesions in the future in patients.

## 2. Materials and Methods

### 2.1. Reagents

All reagents were purchased at Sigma (Munich, Germany) unless otherwise indicated. 4-Styrenesulfonic acid sodium salt hydrate (NaSS) was from Sigma-Aldrich (cat. no. 434574). The anti-SOX9 (C-20) antibody was from Santa Cruz Biotechnology (Heidelberg, Germany), the anti-type-II collagen (AF-5710) and anti-type-I collagen (AF-5610) antibodies from Acris (Hiddenhausen, Germany), and the anti-type-X collagen (COL-10) antibody from Sigma. The biotinylated secondary antibodies and ABC reagent were from Vector Laboratories (Alexis Deutschland GmbH, Grünberg, Germany). The AAVanced Concentration Reagent was from System Bioscience (Heidelberg, Germany) and the Cell Proliferation Reagent WST-1 from Roche Applied Science (Mannheim, Germany).

### 2.2. Bone Marrow Aspirates

The study was approved by the Ethics Committee of the Saarland Physicians Council (*Ärztekammer des Saarlandes*, reference number Ha06/08). All patients provided informed consent before being included in the study that was performed in accordance with the Helsinki Declaration. Bone marrow aspirates (~15 mL; 0.4–1.2 × 10^9^ cells/mL) were prepared from the distal femurs of patients undergoing total knee arthroplasty (*n* = 10, age 73 ± 4 years as the ultimate targets for therapy). Aspirates with MSCs were immediately placed in 96-well plates (150 μL aspirate/well, 6 × 10^7^ cells) and kept at 37 °C [39] until addition of the various films and medium as described below.

### 2.3. Preparation of the Poly(ε-caprolactone) Films

The poly(ε-caprolactone) (PCL) films were prepared by spin-coating as previously described [38]. Briefly, a PCL solution (60% (*w/v*) in dichloromethane) was dropped on a glass slide for spinning (30 sec, 1,500 rpm) with a SPIN150-v3 SPS and the films were air-dried for 2 h and next vacuum-dried for 24 h. The films were then cut in 4-mm disks and some films were grafted with pNaSS (1.3 × 10^−5^ mol/g) via ozonation (10 min, 30 °C). The films were placed in a degassed NaSS solution (15% (*w/v*) in distilled water) for graft polymerization (3 h, 45 °C). The films were then washed using distilled water, 0.15 M NaCl, and PBS and then rinsed for vacuum-drying.

### 2.4. Preparation of the rAAV Vectors

The vectors were prepared using pSSV9, a parental AAV-2 genomic clone [48,49]. rAAV-FLAG-hsox9 carries a 1.7-kb FLAG-tagged human sox9 (hsox9) cDNA sequence controlled by the cytomegalovirus immediate-early (CMV-IE) promoter [39,43]. Conventional packaging of the vectors (not self-complementary) was performed using helper-free (two-plasmid) transfection in 293 cells with the packaging plasmid pXX2 and adenovirus helper plasmid pXX6 [39]. The vectors were then purified via the AAVanced Concentration Reagent. Vector preparations were titered by real-time PCR [39], with titers averaging 10^10^ transgene copies/mL (~1/500 functional recombinant viral particles).

### 2.5. rAAV Vector Immobilization on the PCL Films

The vectors (40 μL, 8 *×* 10^5^ transgene copies) were mixed with 0.002% poly-l-lysine overnight at 37 °C and the samples were next immobilized for 2 h on the films by direct dropping at 37 °C [20,39] to create rAAV-coated PCL films. Some films were also left without rAAV coating as controls. Coating of the films with a reporter vector like rAAV-lacZ [39] was not included here due to a limited access to primary aspirates and as we refer to our work showing that rAAV-lacZ gene transfer via such films does not affect the chondrogenesis of such aspirates [39].

### 2.6. rAAV-Mediated Gene Transfer

Aliquots of human bone marrow aspirates (150 μL, 6 × 10^7^ cells) containing MSCs [39,43] were placed in contact with the rAAV-coated PCL films (MOI = 75) with fibrinogen/thrombin (17 mg/mL/5 U/mL) (Baxter, Volketswil, Switzerland) in 96-well plates. The samples were then either kept in defined chondrogenic differentiation medium (DMEM high glucose 4.5 g/L, 100 U/mL penicillin, 100 μg/mL streptomycin, 0.1 μM dexamethasone, 50 μg/mL ascorbic acid, 40 μg/mL proline, 110 μg/mL pyruvate, 6.25 μg/mL of insulin, 6.25 μg/mL transferrin, 6.25 μg/mL selenious acid, 1.25 μg/mL bovine serum albumin, 5.55 μg/mL linoleic acid, and 10 ng/mL TGF-β3) [39,43] or in osteogenic differentiation medium (StemPro Osteogenesis Differentiation kit with 100 U/mL penicillin, 100 μg/mL streptomycin) [39] (Life Technologies GmbH, Darmstadt, Germany) in a humidified atmosphere with 5% CO_2_ and at 37 °C for up to 21 days for the analyses.

### 2.7. Transgene Expression

Expression of SOX9 was assessed by immunohistochemical analysis with a specific primary antibody, a biotinylated secondary antibody, and using the ABC method with diaminobenzidine (DAB) as a chromogen for monitoring under light microscopy (Olympus BX45; Olympus, Hamburg, Germany) [43].

### 2.8. Biological Analyses

The aspirates with films were harvested with selective papain digestion. The proteoglycan contents were determined by binding to dimethylmethylene blue dye, with values normalized to total cellular proteins monitored via Pierce Thermo Scientific Protein Assay (Thermo Fisher Scientific, Schwerte, Germany) [43]. All measurements were performed using a GENios spectrophotometer/fluorometer (Tecan, Crailsheim, Germany). Cell viability was examined using the Cell Proliferation Reagent WST-1 (OD^450 nm^ proportional to the cell numbers) [39] using a GENios spectrophotomer/fluorometer.

### 2.9. Histology and Immunohistochemistry

The aspirates with films were fixed (4% formalin), dehydrated (graded alcohols), embedded (paraffin), sectioned (3 μm), and stained with hematoxylin and eosin (H&E, cellularity), toluidine blue (matrix proteoglycans), and alizarin red (mineralization) [39,43]. Immunohistochemical analyses were also performed to detect the expression of type-II, -I, and -X collagen using specific primary antibodies, biotinylated secondary antibodies, and the ABC method with DAB [39,43]. Control conditions (lack of primary antibody) were included to check for secondary immunoglobulins. All sections were evaluated under light microscopy (Olympus BX45).

### 2.10. Histomorphometric Analyses

The cell densities (cells/mm^2^ on H&E-stained sections), the percentages of SOX9-positive cells (SOX9 positively-stained cells to the total numbers of cells on immunohistochemical sections), and the intensities of toluidine blue and alizarin red staining and of type-II/-I/-X collagen immunostaining were measured with four immunohistochemical (SOX9, type-II/-I/-X collagen) and histological (H&E, toluidine blue, alizarin red) sections per condition using the SIS AnalySIS program (Olympus), Adobe photoshop (Adobe Systems, Unterschleissheim, Germany), and Scion Image (Scion Corporation, Frederick, MD, USA) [39,43]. Toluidine blue- and alizarin red-stained and type-II/-I/-X collagen-immunostained sections were scored (uniformity, density) using a modified Bern score grading system [39,43] (0 = no staining; 1 = heterogeneous and/or weak staining; 2 = homogeneous and/or moderate staining; 3 = homogeneous and/or intense staining; 4 = very intense staining). Sections were scored blind by two individuals with regard to the conditions.

### 2.11. Real-Time RT-PCR Analysis

Total cellular RNA was extracted with the RNeasy Protect Mini Kit and on-column RNase-free DNase treatment (Qiagen, Hilden, Germany). RNA was eluted in 30 μL RNase-free water and reverse transcription was performed using 8 μL of eluate and the 1st Strand cDNA Synthesis kit for RT-PCR (AMV) (Roche Applied Science). Real-time PCR amplification was performed using 3 μL of cDNA product with Brilliant SYBR Green QPCR Master Mix (Stratagene, Agilent Technologies, Waldbronn, Germany) [39,43] on an Mx3000P QPCR system (Stratagene). The following conditions were used: (10 min at 95 °C), 55 cycles of amplification (30 sec denaturation at 95 °C, 1 min annealing at 55 °C, 30 sec extension at 72 °C), denaturation (1 min at 95 °C), and final incubation (30 sec at 55 °C). The primers (Invitrogen GmbH, Darmstadt, Germany) employed were: SOX9 (chondrogenic marker; forward 5′-ACACACAGCTCACTCGACCTTG-3′; reverse 5′-GGGAATTCTGGTTGGTCCTCT-3′), type-II collagen (COL2A1; chondrogenic marker; forward 5′-GGACTTTTCTCCCCTCTCT-3′; reverse 5′-GACCCGAAGGTCTTACAGGA-3′), aggrecan (ACAN; chondrogenic marker; forward 5′-GAGATGGAGGGTGAGGTC-3′; reverse 5′-ACGCTGCCTCGGGCTTC-3′), type-I collagen (COL1A1; osteogenic marker; forward 5′-ACGTCCTGGTGAAGTTGGTC-3′; reverse 5′-ACCAGGGAAGCCTCTCTCTC-3′), type-X collagen (COL10A1; marker of hypertrophy; forward 5′-CCCTCTTGTTAGTGCCAACC-3′; reverse 5′-AGATTCCAGTCCTTGGGTCA-3′), and glyceraldehyde-3-phosphate dehydrogenase (GAPDH; housekeeping gene and internal control; forward, 5′-GAAGGTGAAGGTCGGAGTC-3′; reverse, 5′-GAAGATGGTGATGGGATTTC-3′) (all 150 nM final concentration) [39,43]. Control conditions included reactions with water and non-reverse-transcribed mRNA and product specificity was confirmed by melting curve analysis and agarose gel electrophoresis. The threshold cycle (Ct) value for each gene was obtained for each amplification with the MxPro QPCR software (Stratagene). Values were normalized to GAPDH expression with the 2^−ΔΔCt^ method [39,43].

### 2.12. Statistical Analysis

Data are provided as mean ± standard deviation (SD) of separate experiments. Each condition was performed in triplicate in three independent experiments per patient, using all patients for all experiments. Data were obtained by two individuals blinded with respect to the groups. The t test and the Mann-Whitney Rank Sum Test were used where appropriate. A P value of less than 0.05 was considered statistically significant.

## 3. Results

### 3.1. sox9/pNaSS-Grafted PCL Films Promote the Effective Overexpression of SOX9 in Human Bone Marrow Aspirates

The candidate rAAV-FLAG-hsox9 vector was first coated on poly(sodium styrene sulfonate) (pNaSS)-grafted versus ungrafted poly(*ε*-caprolactone) (PCL) films (sox9/pNaSS-grafted PCL and sox9/ungrafted PCL, respectively) in order to examine the ability of the systems to promote SOX9 expression in human bone marrow aspirates over time relative to other treatments (pNaSS-grafted and ungrafted PCL films without vector coating, i.e., no vector/pNaSS-grafted PCL and no vector/ungrafted PCL, respectively) [39].

**Figure 1:**
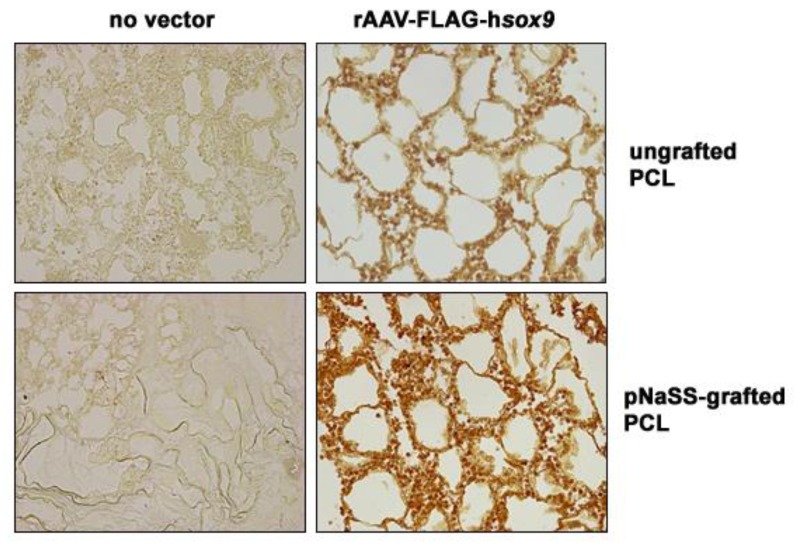
Effective sex-determining region Y-type high-mobility group box 9 (SOX9) overexpression in human bone marrow aspirates transduced with recombinant adeno-associated virus (rAAV) *sox9*-coated poly(ε-caprolactone) (PCL) films. poly(sodium styrene sulfonate) (pNaSS)-grafted and ungrafted PCL films were coated with rAAV-FLAG-h*sox9* or let without vector treatment prior to incubation with the aspirates and SOX9 expression was immunodetected after 21 days (magnification ×20; all representative data).

Effective rAAV-mediated SOX9 overexpression was achieved in human bone marrow aspirates receiving the sox9/pNaSS-grafted or sox9/ungrafted PCL films after 21 days in chondrogenic medium as seen by significantly more elevated levels of SOX9 production compared with aspirates where films lacking vector coating were provided (up to 16.9-fold difference in the % of SOX9-positive cells, P ≤ 0.001) (Figure 1 and Table 1). Interestingly, the levels of SOX9 overexpression were more elevated when using sox9/pNaSS-grafted films than with sox9/ungrafted films (1.2-fold difference in the % of SOX9-positive cells, P ≤ 0.001) (Figure 1 and Table 1).

### 3.2. sox9/pNaSS-Grafted PCL Films Induce Proteoglycan and Type-II Collagen Deposition in Human Bone Marrow Aspirates

The candidate rAAV-FLAG-hsox9 vector coated on pNaSS-grafted versus ungrafted PCL films was then delivered to human bone marrow aspirates in order to examine the ability of the systems to activate the biological and chondrogenic processes in human bone marrow aspirates over time relative to other treatments.

**Figure 2:**
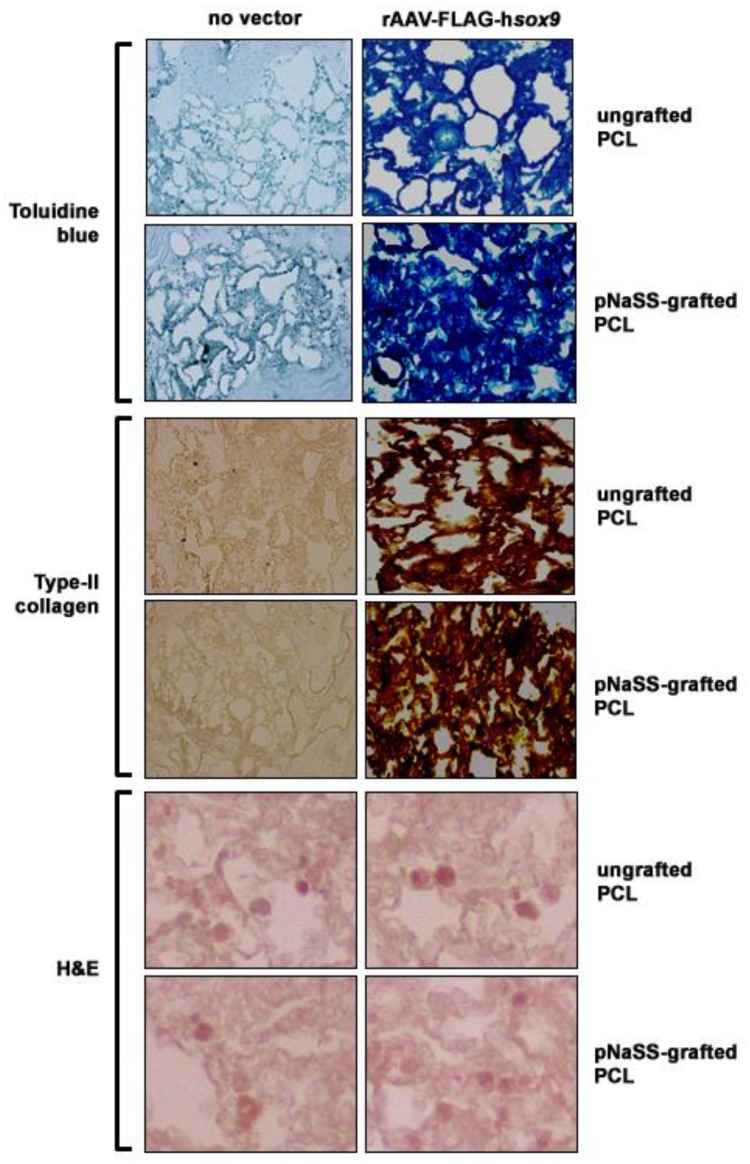
Induction of proteoglycan and type-II collagen deposition in human bone marrow aspirates transduced with rAAV *sox9*-coated PCL films. pNaSS-grafted and ungrafted PCL films were coated with rAAV-FLAG-h*sox9* or let without vector treatment prior to incubation with the aspirates and matrix proteoglycans (toluidine blue), type-II collagen immunoexpression, and cellularity (H&E) were evaluated after 21 days (magnification ×20 except for H&E at magnification ×40; all representative data).

Administration of rAAV-FLAG-hsox9 via pNaSS-grafted and ungrafted PCL films to the aspirates led to a higher deposition of proteoglycans after 21 days in chondrogenic medium as noted by more intense toluidine blue staining with these systems compared with other conditions (up to 2.8-fold difference, P ≤ 0.001), with an even more potent effect when applying pNaSS-grafted PCL films (1.2-fold difference versus ungrafted films, P = 0.015) (Figure 2 and Table 1). These observations were corroborated by an analysis of the proteoglycan contents in the aspirates, showing higher amounts produced with either sox9/pNaSS-grafted or sox9/ungrafted PCL films (up to 2.1-fold difference versus other conditions, P ≤ 0.001), especially upon pNaSS grafting (1.2-fold difference versus ungrafted films, P ≤ 0.001) (Table 1). Similar results were obtained when analyzing the deposition of type-II collagen, with more intense type-II collagen immunostaining with sox9/pNaSS-grafted or sox9/ungrafted PCL films (up to 13-fold difference versus other conditions, P ≤ 0.001) and more potent effects with pNaSS-grafted PCL films (1.1-fold difference versus ungrafted films, P = 0.039) (Figure 2 and Table 1). In marked contrast, no effects of rAAV sox9 gene transfer via pNaSS-grafted and ungrafted PCL films were noted on the levels of cell proliferation in the aspirates versus other treatments (cell densities: P ≥ 0.104; WST-1 assay: P ≥ 0.083), regardless of the type of film employed (cell densities: P ≥ 0.147; WST-1 assay: P = 0.096) (Figure 2 and Table 1).

### 3.3. sox9/pNaSS-Grafted PCL Films Reduce Mineralization and Type-I and -X Collagen Deposition in Human Bone Marrow Aspirates

The candidate rAAV-FLAG-hsox9 vector coated on pNaSS-grafted versus ungrafted PCL films was then delivered to human bone marrow aspirates in order to test the ability of these systems to restrict premature osteogenic and hypertrophic differentiation in human bone marrow aspirates over time relative to other treatments.

**Figure 3:**
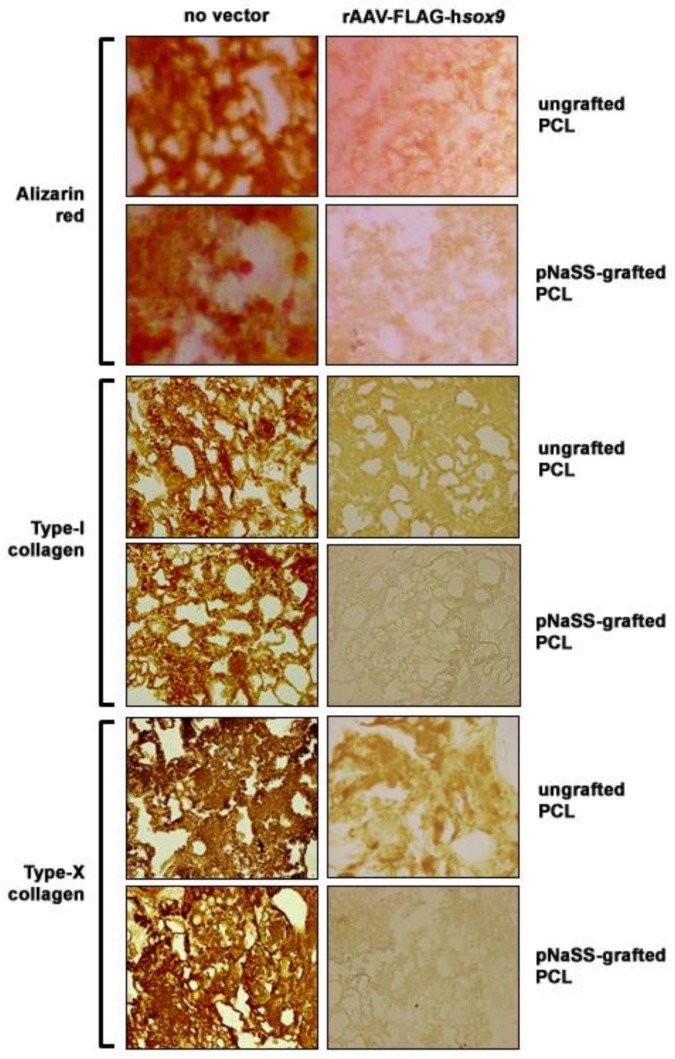
Reduction of mineralization and type-I and -X collagen deposition in human bone marrow aspirates transduced with rAAV *sox9*-coated PCL films. pNaSS-grafted and ungrafted PCL films were coated with rAAV-FLAG-h*sox9* or let without vector treatment prior to incubation with the aspirates and mineralization (alizarin red) and type-I and -X collagen immunoexpression were evaluated after 21 days (magnification ×20; all representative data).

Application of rAAV-FLAG-hsox9 via pNaSS-grafted and ungrafted PCL films to the aspirates led to lesser matrix mineralization after 21 days in osteogenic medium as noted by decreased alizarin red staining with these systems compared with other conditions (up to 4.3-fold difference, P ≤ 0.001), with an even more potent effect when using pNaSS-grafted PCL films (2.1-fold difference versus ungrafted films, P ≤ 0.001) (Figure 3 and Table 1). Similar results were obtained when analyzing the deposition of osteogenic type-I collagen and hypertrophic type-X collagen, with reduced type-I and -X collagen immunostaining with sox9/pNaSS-grafted or sox9/ungrafted PCL films (type-I collagen: up to 5.3-fold difference versus other conditions, P ≤ 0.001; type-X collagen: up to 6.5-fold difference versus other conditions, P ≤ 0.001), with even more potent effects when using pNaSS-grafted PCL films (type-I collagen: 1.9-fold difference versus ungrafted films, P = 0.015; type-X collagen: 2.2-fold difference versus ungrafted films, P = 0.032) (Figure 3 and Table 1).

### 3.4. sox9/pNaSS-Grafted PCL Films Activate the Chondrogenic Expression Profiles and Reduce Osteogenic and Hypertrophic Expression in Human Bone Marrow Aspirates

**Figure 4:**
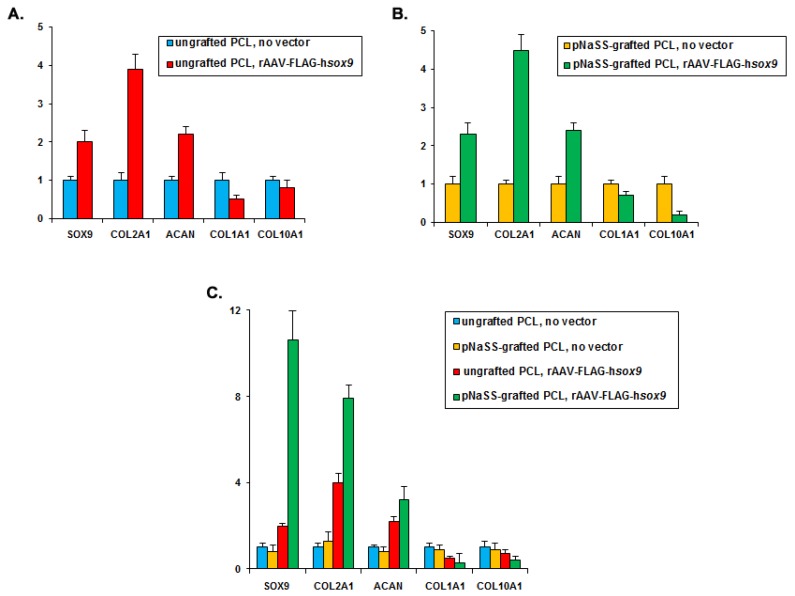
Induction of chondrogenic expression profiles and reduction of osteogenic and hypertrophic expression in human bone marrow aspirates transduced with rAAV *sox9*-coated PCL films. pNaSS-grafted and ungrafted PCL films were coated with rAAV-FLAG-h*sox9* or let without vector treatment prior to incubation with the aspirates and the profiles of SOX9, COL2A1, ACAN, COL1A1, and COL10A1 *versus* GAPDH were evaluated by real-time RT-PCR after 21 days ((**A**,**B**): fold inductions relative to samples receiving each respective PCL films without rAAV; (**C**): fold inductions relative to samples receiving ungrafted PCL films without rAAV; data from all patients).

The previous results were corroborated by a real-time RT-PCR analysis performed in human bone marrow aspirates targeted over time by the candidate rAAV-FLAG-hsox9 vector coated on pNaSS-grafted versus ungrafted PCL films and relative to other treatments. Specifically, administration of rAAV-FLAG-hsox9 via pNaSS-grafted and ungrafted PCL films to the aspirates compared with each respective control condition led to higher levels of chondrogenic SOX9, COL2A1, and ACAN gene expression profiles in chondrogenic medium (2-, 3.9-, and 2.2-fold difference, respectively, in the sox9/ungrafted versus ungrafted PCL films and 2.3-, 4.5-, and 2.4-fold difference, respectively, in the sox9/pNaSS-grafted versus pNaSS-grafted PCL films, always P ≤ 0.001) (Figure 4A,B), with even more potent effects when applying pNaSS-grafted PCL films (6-, 1.8-, and 1.4-fold difference, respectively, in the sox9/pNaSS-grafted versus sox9/ungrafted PCL films, always P ≤ 0.001) (Figure 4C). Also, application of rAAV-FLAG-hsox9 via pNaSS-grafted and ungrafted PCL films to the aspirates compared with each respective control condition led to lower levels of osteogenic COL1A1 and hypertrophic COL10A1 gene expression profiles in osteogenic medium (2- and 1.3-fold difference, respectively, in the sox9/ungrafted versus ungrafted PCL films and 1.4- and 5-fold difference, respectively, in the sox9/pNaSS-grafted versus pNaSS-grafted PCL films, always P ≤ 0.001) (Figure 4A,B), with even more potent effects when applying pNaSS-grafted PCL films (2.3- and 3.5-fold difference, respectively, in the sox9/pNaSS-grafted versus sox9/ungrafted PCL films, always P ≤ 0.001) (Figure 4C).

## 4. Discussion

Scaffold-assisted gene therapy is a powerful tool to durably and safely enhance the processes of cartilage repair [10,11,12] via single-step, controlled delivery of clinically adapted rAAV gene transfer vectors [25,26,27,28,29,30,31,32,33]. In the current work, we tested the ability of mechanically stable, biocompatible PCL scaffolds functionalized with a bioactive pNaSS molecule to transfer rAAV vectors coding for the cartilage-specific SOX9 transcription factor to human bone marrow aspirates as a means to generate more efficient, non-invasive systems to treat focal cartilage lesions compared with a less stable scaffold-free vector application lacking scaffolding benefits for the cell targets [42] or with complex, indirect transplantation of rAAV-modified aspirates via such materials [43].

The present findings first indicate that PCL films are capable of promoting the successful overexpression of SOX9 via delivery of coated rAAV vectors to human bone marrow aspirates over time (21 days), most particularly following grafting of the films with pNaSS (up to 94.4% transduction efficiencies) compared with other conditions (pNaSS-grafted and ungrafted PCL films without vector coating), expanding work in aspirate samples modified by free rAAV SOX9 gene transfer [42] that may further be seeded in PCL scaffolds for indirect therapeutic applications [43].

The data next reveal that efficient, rAAV-mediated overexpression of SOX9 via PCL-guided gene delivery allowed to stimulate the chondrogenic differentiation activities (proteoglycan and type-II collagen expression) in the aspirates over time (21 days) relative to the other treatments, in good agreement with the properties of the transcription factor [40] and with our previous findings using direct [42] and indirect [43] gene transfer of the current construct to target such samples. Of particular interest, these beneficial effects were more pronounced when using pNaSS-grafted PCL films to deliver the vectors to their targets, probably due to the superior ability of these systems to transfer the rAAV SOX9 construct, supporting the concept of manipulating such functionalized material for improved translational applications. Interestingly, the levels of cell proliferation in the aspirates were not affected by SOX9 gene transfer regardless of the type of films utilized, as also noted when directly providing the same rAAV SOX9 vector to human bone marrow aspirates prior to seeding in woven PCL scaffolds [43], again concordant with the properties of SOX9 [40].

Equally important, application of rAAV SOX9 via PCL films allowed to limit undesirable osteogenic and hypertrophic differentiation events (matrix mineralization, type-I and -X expression) in the aspirates over time *versus* other treatments, especially when using pNaSS-grafted PCL films, again reflecting the higher capability of these systems to deliver rAAV, consistent with our work in direct (free rAAV SOX9 vector) gene transfer conditions [42] or when indirectly placing rAAV SOX9-transduced aspirates within woven PCL scaffolds [43] and in agreement with the effects of SOX9 [50].

Overall, the current study shows the potential of delivering a therapeutic rAAV (SOX9) gene vector to human bone marrow aspirates using PCL films functionalized by pNaSS grafting as an innovative, convenient system to provide effective, off-the-shelf tools for applications that aim at improving cartilage repair. Work is ongoing to further test the value of the approach in vivo using relevant (orthotopic) models of cartilage lesions [14,16,51] and the additional benefits of the scaffold-guided strategy *versus* direct rAAV SOX9 injection [51], especially in light of potentially deleterious immune responses against rAAV gene transfer in vivo [24].

## 5. Conclusions

This study demonstrates the possibility of applying therapeutic sequences via solid scaffold-guided gene transfer in chondroreparative marrow aspirates as translational platforms to readily treat sites of cartilage injury in individuals in the near future.

## Figures and Tables

**Table 1:** Histomorphometric and biological analyses in human bone marrow aspirates transduced with the rAAV-coated PCL films.

Parameters	No Vector/Ungrafted PCL	No Vector/pNaSS-grafted PCL	*sox9*/Ungrafted PCL	*sox9*/pNaSS-Grafted PCL
SOX9	5.6 ± 2.7	6.1 ± 2.9	78.6 ± 2.4 ^a,b^	94.4 ± 2.2 ^a,b,c^
Toluidine blue	1.4 ± 0.5	1.6 ± 0.5	3.3 ± 0.5 ^a,b^	3.9 ± 0.4 ^a,b,c^
Proteoglycans	0.040 ± 0.002	0.039 ± 0.002	0.070 ± 0.003 ^a,b^	0.083 ± 0.001 ^a,b,c^
Type-II collagen	0.3 ± 0.5	0.4 ± 0.5	3.4 ± 0.5 ^a,b^	3.9 ± 0.4 ^a,b,c^
Cell densities	2481 ± 57	2523 ± 72	2520 ± 53	2515 ± 58
WST-1	0.51 ± 0.07	0.63 ± 0.12	0.61 ± 0.10	0.70 ± 0.07
Alizarin red	3.7 ± 0.5	3.9 ± 0.4	1.9 ± 0.4 ^a,b^	0.9 ± 0.4 ^a,b,c^
Type-I collagen	3.7 ± 0.5	3.7 ± 0.5	1.3 ± 0.5 ^a,b^	0.7 ± 0.5 ^a,b,c^
Type-X collagen	3.9 ± 0.4	3.9 ± 0.4	1.3 ± 0.8 ^a,b^	0.6 ± 0.5 ^a,b,c^

SOX9 expression is in % of positively-stained cells to the total numbers of cells on immunohistochemical sections. The type-II/-I/-X collagen-immunostained and the toluidine blue- and alizarin red-stained sections were scored using a modified Bern score grading system [39,43] (0 = no staining, 1 = heterogeneous and/or weak staining, 2 = homogeneous and/or moderate staining, 3 = homogeneous and/or intense staining, 4 = very intense staining). The proteoglycan contents are in μg/μg total proteins. The cell densities on H&E-stained sections are in cells/mm^2^. The data of the WST-1 assay are given as OD^450 nm^. Values are given as mean ± standard deviation (SD). Statistically significant relative to ^a^ no vector/ungrafted PCL, ^b^ no vector/pNaSS-grafted PCL, and ^c^ *sox9*/ungrafted PCL.
